# Optimization of the Danish National Electronic Prescribing System to Improve Patient Safety: Development of a User-Friendly Prototype of the Digital Platform Shared Medication Record

**DOI:** 10.3390/pharmacy11020041

**Published:** 2023-02-22

**Authors:** Anissa Aharaz, Cecillie Louise Kejser, Mille Wilhjelm Poulsen, Sara Jeftic, Aisha Isabella Ulstrup-Hansen, Lillian Mørch Jørgensen, Esben Iversen, Anne Mette Thorhauge, Morten Baltzer Houlind

**Affiliations:** 1 The Capital Region Pharmacy, 2730 Herlev, Denmark; 2 Department of Clinical Research, Copenhagen University Hospital–Amager and Hvidovre, 2650 Copenhagen, Denmark; 3 Department of Communication, University of Copenhagen, 2300 Copenhagen, Denmark; 4 Emergency Department, Copenhagen University Hospital–Amager and Hvidovre, Kettegård Alle 30, 2650 Hvidovre, Denmark; 5 Department of Drug Design and Pharmacology, University of Copenhagen, 2100 Copenhagen, Denmark

**Keywords:** digitalization, healthcare sector, sector transition, patient safety, medication error, multimorbidity

## Abstract

This study uses a participatory design to develop a user-friendly prototype of the current Danish digital platform, Shared Medication Record (SMR), to improve patient safety and minimize medication errors for patients with multimorbidity. A fundamental challenge for medication prescribing is the lack of access to an accurate medication list, which impairs effective communication between healthcare professionals and increases the risk of medication errors. We used a participatory design to identify the major problems with the existing SMR and develop a prototype for a redesigned SMR that addresses these problems. We argue that this prototype will improve communication between healthcare providers, promote patient involvement in their own care, and ultimately reduce medication errors related to the SMR. Moreover, we argue that the participatory design with its emphasis on user involvement and design iterations is a strong approach when designing IT solutions for complex problems in healthcare.

## 1. Introduction

A fundamental challenge for healthcare providers is the lack of access to reliable information regarding patients’ actual prescribed medications, which prevents providers from delivering optimal services and increases the risk of medication errors [1,2,3,4]. This is particularly relevant for patients with multimorbidity who are characterized by a high prevalence of polypharmacy, inappropriate medications, and adverse drug events [5,6,7,8]. Medication reconciliation and medication review are conducted in most acute settings within Denmark to deal with these problems, but discrepancies in patients’ actual medication lists remain common [9,10,11]. The digitalization of medical records has the potential to increase the efficiency and safety of medication prescribing by centralizing medication data from diverse healthcare sectors and accommodating movement by patients between sectors [4].

Several countries have already developed or are developing digital platforms that can be accessed by patients, caregivers, and healthcare providers. In Denmark, this digital platform is known as the Shared Medication Record (SMR). The SMR was developed in 2011 and fully integrated into the existing electronic medical record by 2015 [11,12]. According to the Danish Health Data Authority, the purpose of the SMR is to reduce medication errors, hospital readmissions caused by these medication errors, and the time required to clarify patients’ medication use [13,14]. The SMR has extensive functionalities to cover all aspects of medication prescribing, but the presentation of these functionalities is often inefficient and does not always align with the normal workflow. As a result, medication records are not continually updated, and the SMR has not been shown to improve medication reconciliation across healthcare sectors [9,11,15,16].

A report by the Danish Health Data Authority showed that the SMR is updated less frequently in outpatient settings compared to inpatient settings [16]. The report also revealed that no regions within Denmark have implemented the SMR to fulfill the requirement that medication records should be updated every time a medication order is started or changed [16]. Without consistent updates, the SMR becomes unreliable and makes it challenging to obtain an accurate overview of a patient’s medication list, which can ultimately lead to medication errors. The Danish Patient Safety Authority showed a 14% increase in the number of adverse drug events reported from 2018 to 2019, possibly emphasizing the shortcomings of the SMR in its current state [17,18].

Healthcare systems are sometimes developed using participatory design (PD), which involves direct and continuous user inputs by considering their perceptions of technology [19,20]. One important feature of PD is iteration, which is the process of identifying challenges, addressing those challenges, and then identifying new challenges [21]. In this way, each iteration builds on the information learned from the previous iterations [22]. Another important feature of PD is interviews, which are a qualitative approach to understanding the perspectives from different users of the system [23]. The information collected from interviews can provide insights into which aspects of the system users see as relevant, which can help to improve future iterations of the system. In this study, we utilize a PD to examine the issues with the current SMR within a Danish hospital and develop a prototype that addresses these issues. Our specific aims are to optimize the SMR interface and reduce the risk of medication errors.

## 2. Methods

### 2.1. Shared Medication Record

The SMR is a central database managed by the Danish Health Data Authority containing information about all medications prescribed and purchased by Danish citizens within the past two years [24]. The SMR is accessible by all members of a patient’s healthcare team and provides information regarding medications, vaccinations, medical grant applications, outpatient orders, and authorizations. Physicians and dentists have access to all actions within the SMR (including adding, removing, or changing medication prescriptions), while all other clinicians, nurses, and pharmacy staff that are in daily contact with patients have limited access [24,25].

Creating a new prescription in the SMR requires the following steps: (A) log in; (B) enter the front page; (C) locate the patient’s record; (D) navigate to the medication information; if it is a prescription for a new medication, then (E) click on ‘create a prescription’ and (F) fill in the relevant medication information; if it is a prescription for an existing medication, then (G) click on ‘current prescriptions’, (H) ‘prescription’, and (E) fill in the relevant medication information in the ‘create a prescription’ page [22]. See Appendix A for an illustration of the different steps.

### 2.2. Setting

This study was conducted at Copenhagen University Hospital, Hvidovre, Denmark (hereafter: Hvidovre Hospital). Hvidovre Hospital has approximately 3000 employees and covers approximately 550,000 citizens from 10 municipalities. Each year, the hospital has approximately 14,000 medical admissions of which 85% are acute admissions. All medication information from the primary sector that comes through the SMR during admission requires a pharmacist to obtain the actual patient medication list to avoid medication errors during hospitalization.

### 2.3. Design and Participants

The purpose of this study was to develop a user-friendly prototype of the SMR at Hvidovre Hospital. To do this, semi-structured interviews with SMR users (healthcare professionals and patients) were combined with an iterative PD approach to gain insight into users’ daily work practice and experience with the SMR. Since the current SMR is a major challenge in both inpatient and outpatient settings, we included healthcare professionals from both of these settings [16]. Healthcare professionals were selected from three medical specialties (endocrinology, cardiology, and emergency medicine) and were prioritized with the goal of obtaining diverse professional backgrounds, specialties, roles, and responsibilities related to the medication prescribing process. In total, nine healthcare professionals were selected for interview: three physicians, three nurses, two pharmacists, and one pharmacy technician. Since patients are also users of the SMR, two patients were interviewed to obtain patient perspectives. Both patients used hyperpolypharmacy (≥10 medications) and self-dispensed and self-administered their medications at home.

### 2.4. Semi-Structured Interview

All interviews were conducted at Hvidovre Hospital in separate rooms by one of the study authors. Interviews were semi-structured to adapt each interview to the user’s specific experiences and adjust the content of the interviews based on new challenges that emerged during the study [22]. Semi-structured interviews are commonly used in healthcare to explore unique experiences and gather subjective views on sensitive topics [26]. For this particular study, the interviews were developed and conducted by five qualified students in information technology (IT) and quality assurance (QA). Each interview was scheduled to last 30 min and addressed the following questions:

- What is the role of the SMR in your work?
- What are your experiences with the SMR?
- Do you have any suggestions for how to improve the SMR functionality?

Interview responses were analyzed to identify keywords representing the most common challenges with the SMR. This process included initial transcription, read-through of the initial transcripts, revision of the transcripts, determination of keywords, and revision of keywords [23]. Abbreviated transcripts and keywords from each interview are shown in Appendix B and Appendix C. Keywords were then classified into themes, and thematic analysis was used to select the themes critical to implementation of the SMR [27]. This type of data analysis gave the research the opportunity to explore what was similar or different on a certain subject [27].

### 2.5. Iterative Design

**Figure 1:**
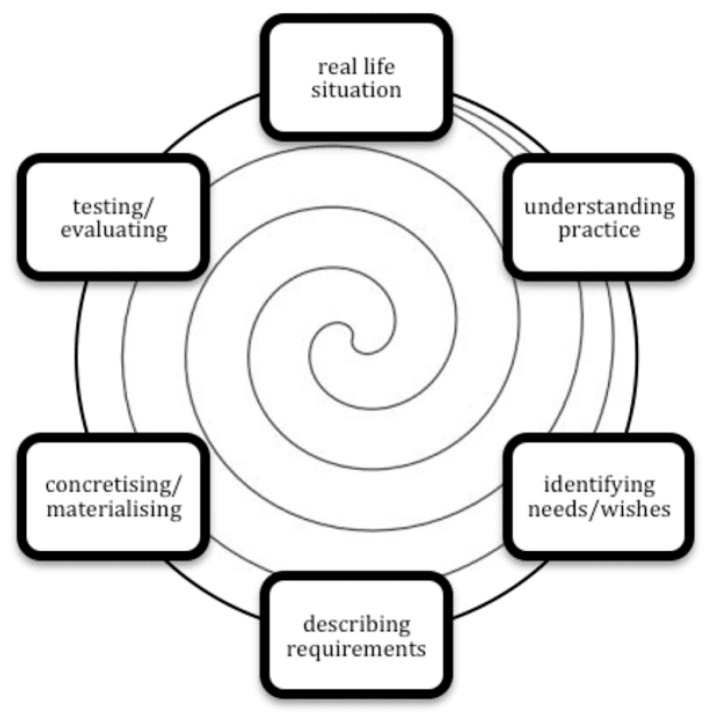
Conceptual model of the iterative design process [20].

Once major themes were identified by the interviews, an iterative design was used to develop and optimize a prototype for a new SMR [20]. The conceptual model for this iterative design involved six phases in the conceptual model, as shown in Figure 1.

After the first iteration, feedback on the initial SMR prototype was collected from five healthcare professionals representing different aspects of the healthcare team. This feedback formed the basis for the second iteration of the SMR prototype, which was again presented to the same participants for feedback and used to develop a final version of the SMR prototype.

## 3. Results

### 3.1. Themes

Analysis of the interviews identified five major themes, which are represented by the following key citations (see Annex 2 for a full overview):

1. **The SMR does not get updated regularly**

*“It is a requirement that the patients get a medical note. We can’t even print this note because the doctors technically have not approved it in SMR. This affects us and makes it difficult for us to do our jobs.”*

2. **It is unclear who has the responsibility to update the SMR**

*“I think that a part of the issue is that the responsibility is complex in some matters. I mean, you need to consider, when a patient gets prescribed medicine, it is usually not just one person, but several medical professions who is a part of this process...”*

3. **The system is complex, time-consuming, and unintuitive**

*“The thing with SMR as is that the concept works well, and it is really handy that it is available on every device. The problem with SMR is the integration with the health platform. This is horrible.”*

4. **Patients are often unaware that they can access the SMR, so they do not have complete insight into their medication**

*“Not a lot of those who use SMR have an overview over their medicine. It is rare that I experience people using it—including the younger patients.”*

5. **There is a need for other professional groups and/or patients to be involved in the prescribing process**

*“It may be linked to age and tech flair, but I think a lot of patients will benefit from more involvement... I think it could create dialogue and be easier for doctors to clean up old medicine or update the SMR.”*

Several themes appear to be interrelated. For example, the irregularity of updates (theme 1) is likely a consequence of deferred responsibility (theme 2), overcomplexity (theme 3), limited patient knowledge (theme 4), and ineffective communication (theme 5). Patient safety and workflow efficiency were identified as the two primary consequences of an ineffective digital platform. If medication records are not kept up-to-date, then the information on the platform becomes unreliable. For patients, this may decrease trust in the healthcare system or lead to reliance on outdated information. For healthcare providers, it may increase the time required to determine what medications have been prescribed. Based on these observations, adjustments to the existing SMR were used to create a prototype for an optimized SMR that would improve both patient safety and workflow efficiency.

### 3.2. Prototype for an Optimized SMR

**Figure 2:**
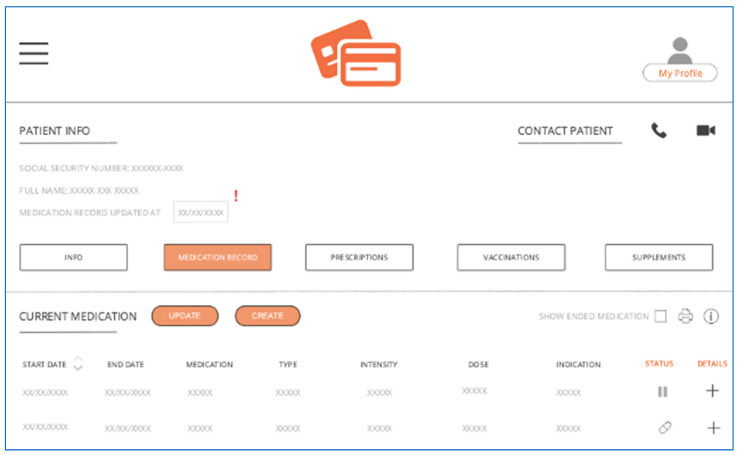
Illustration of a redesign of the Shared Medication Record.

The complete SMR prototype (in Danish) is shown in Appendix D. An abbreviated version (in English) is shown in Figure 2, and an overview of the proposed features is shown in Table 1. The main features of the prototype include a redesigned interface, new functions, and simplified buttons. The redesigned interface aims to make the SMR more manageable, navigable, and straightforward for healthcare professionals. The ‘last updated’ function aims to increase transparency about when the last update was performed, while the ‘edit’, ’renew’, and ’delete’ buttons aim to decrease errors related to incorrect or duplicate prescriptions. During the final interviews, study participants agreed that the prototype provides a more transparent overview of patients’ medications.

## 4. Discussion

### 4.1. Patient Safety

Optimizing digital platforms for use across healthcare sectors requires a basic understanding of each sector and the interactions between them. In the Danish healthcare system, each sector is responsible for different aspects of patient care, yet there is limited access to patient data from other sectors. Therefore, comprehensive patient care across sectors requires flexible and effective communication. Digital platforms, such as the SMR, were developed to accommodate these requirements. However, this study reveals some of the limitations of relying on digital platforms that do not have clear guidelines for their use. For example, many participants included in the design process were unsure whether the responsibility to update medication records lies with the general practitioners or hospital physicians. Similar uncertainties have been described by Hammar et al. [3] and Manskow et al. [28]. This professional “stalemate” can lead to incomplete medication lists, cross-sectoral prescribing errors, and adverse drug events [29,30]. Patients with polypharmacy are at particularly high risk of medication errors, in part because they lack insight into their medications and trust that their medical information is handled appropriately [31]. This observation is supported by our interviews with patients, who stated they did not know they could access the SMR.

Effective communication between healthcare professionals is supported by standardization of work tasks. Coordination of these work tasks between sectors is essential for improving workflow and minimizing the time spent on clarifying responsibilities [32]. We believe that our optimized SMR can achieve this coordination by improving functionality and giving clear instructions about users’ responsibilities. Our proposed solution also digitalizes the process of medication reconciliation, which is currently lacking in acute settings within Denmark.

### 4.2. Workflow Efficiency

Our SMR prototype was developed using an iterative participatory design. During each step, we considered who the end-users of the SMR are, what each of their individual needs would be, and how the SMR could be optimized to meet those needs. Most of the participants included in the design process stated that they supported the idea of the SMR, but that practical challenges limited functionality of the SMR in its current state. A common complaint was that the current SMR design is cumbersome and difficult to navigate. Due to the confusing interface, many users stated that they delay their updates or simply skip them altogether, which reduces the reliability of information for other users. Based on these comments, we determined that it was necessary to redesign the SMR to improve workflow efficiency.

Our SMR prototype facilitates dialogue among healthcare professionals across sectors as well as with patients. The redesigned interface gives a better overview of the various information contained within each patient’s medication records, and the new functionalities direct users to the most important information. We believe these changes will increase workflow efficiency for healthcare professionals by streamlining the user experience and enhancing cooperation between healthcare sectors. We have previously demonstrated that interdisciplinary collaboration improves the effectiveness of deprescribing interventions among patients with polypharmacy [33,34]. The simplified buttons in our SMR prototype make it much easier for clinicians across healthcare sectors to deprescribe inappropriate medications. By extension, we expect these changes will promote patient involvement. The new interface makes it easier for patients to understand their medication list and exchange messages with their healthcare team about medication preferences, side effects, and errors. More effective communication via the SMR is expected to reduce the time required for medication reconciliation in the hospital and, ultimately, reduce the number of medication errors [35].

### 4.3. Healthcare Costs

Finally, the redesigned SMR prototype promises to reduce healthcare costs. The digital platform is already functional across Denmark, and a software update would be quick and inexpensive to implement. A more efficient workflow would allow healthcare professionals to spend time on other tasks, and fewer medication errors would lead to fewer readmissions (and all costs related to readmission). This conclusion is supported by Bates et al., who argue that information technology can decrease the frequency of excessive drug doses and lead to substantially lower healthcare costs [36].

### 4.4. Strength and Limitations

In this pilot study, we explored gaps in the digital platform for medication prescribing in a hospital setting. The strength of this study is that the SMR already exists as a digital platform across Denmark, and our prototype for an updated SMR would require a simple software update. The main limitation of our study is that interviewee participation was limited to the hospital setting. During the iterative design process, we specifically selected healthcare professionals that were representative of different professional groups, but future studies are needed to determine the prototype’s effectiveness in other healthcare sectors, such as general practice and home care. We also interviewed two patients to obtain their perspectives, but the prototype should be tested on a wider population of patients with polypharmacy and multimorbidity. Our study was also restricted to Denmark, so research in other countries is necessary to generalize our findings to other patient groups and geographical contexts. A systematic comparison to other healthcare systems would also be necessary to evaluate if the same needs apply to patients with multimorbidity and polypharmacy in general. The PD approach used in this study also has important limitations. This approach enabled us to disentangle complex problems and identify solutions in an ongoing dialogue with a diverse group of end-users. However, PD is built on an assumption of consensus between all end-users, yet conflicting interests between end-users are difficult to address with PD solutions. Moreover, PD is a relatively time-consuming approach, which limits its effectiveness in healthcare systems characterized by limited resources. Finally, we did not test the clinical impact of the prototype.

## 5. Conclusions

This study explored challenges associated with the existing digital platform for medication prescribing within Denmark. Through a PD approach, we identified patient safety and workflow efficiency as the two main challenges for the SMR. We then created a prototype for an updated SMR that would streamline the user experience and improve communication between patients and healthcare professionals across sectors. Ultimately, the goal of these changes is to improve the accuracy of medication lists and reduce medication errors. Future large-scale studies should attempt to replicate our findings in a more diverse patient population and test the clinical impact of an updated prototype.

## Figures and Tables

**Table 1:** An overview of the proposed features for an improved Shared Medication Record. The functions/features marked with (*) are currently implemented in the Shared Medication Record but have been modified in some way.

Implementation	Description
Overall redesign	A more manageable, navigable, and straightforward interface that facilitates up-to-date, complete, and accurate medication records as well as knowledge and information sharing
‘Last updated’-field (A)	States when the medication record was last updated. A notification will appear if it has not been updated for X-months
‘Edit’-button (B)	Current medication can be edited so that the risk of double/incorrect prescriptions is reduced
‘Renew’-button (C)	Current medication can be renewed so that the risk of double prescriptions is reduced *
‘Delete’-button (D)	Current medication can be deleted so that the risk of double/incorrect prescriptions is reduced
‘Note’-button (E)	It makes it possible to write notes to a specific medication (if important, it is indicated with a red exclamation mark)
‘Send notification’-button (F)	It makes it possible to notify a patient’s general practitioner or other relevant parties about any changes, suggestions, notes, etc. in medication
‘Attach file/document’-button (G)	It makes it possible to attach relevant files/pictures/documents in connection with the ‘send notification’ and/or ’note’-button
‘End date’-field (H)	End date requirements in connection with the creation of prescriptions *
‘Medication is about to expire’-function (I)	A notification will appear as the medication is about to expire. If the user does not renew the medication, it will be removed from the ‘current medication’ list and then appear on the list of ‘completed medication.’
‘Irregularities in effectuation’-function (J)	A notification will appear if there are irregularities in the effectuated medication
‘Contact patient’-button (K)	It makes it possible to contact the patient directly via the SMR, either per phone or virtually (with video)
‘Dropdown navigation menu’ (L)	The dropdown navigation menu for more straightforward navigation
‘Add shortcut’-button (M)	Personalized frontpage that lets users select and add the most frequently used shortcuts to the frontpage

**Figure A1:**
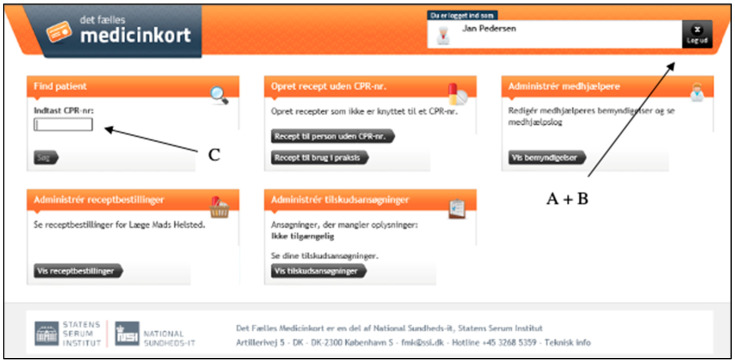
Front page of the Shared Medication record (SMR) patient data entry. A log in; B enter the front page; C locate the patient’s record.

**Figure A2:**
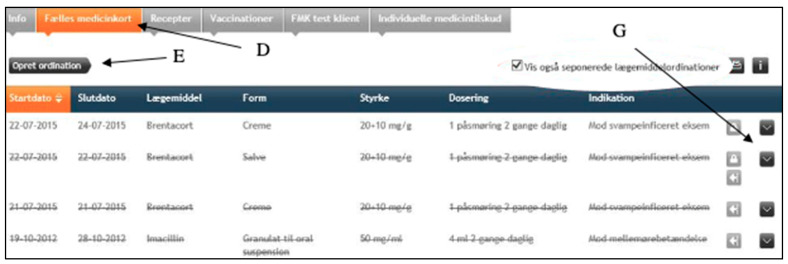

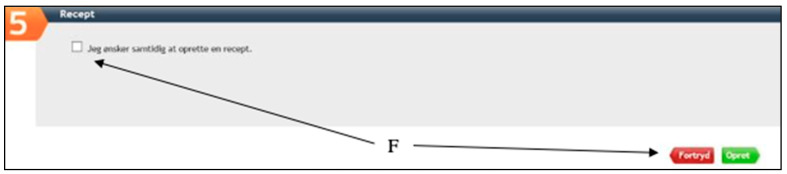
First tab after entry is the patient’s medication list overview. D navigate to medication information if it is a prescription; if it is a new medication, then E click on ‘create a prescription’ and F fill in the relevant medication information; if it is a prescription for an existing medication, then G click on ‘current prescriptions’.

**Figure A3:**
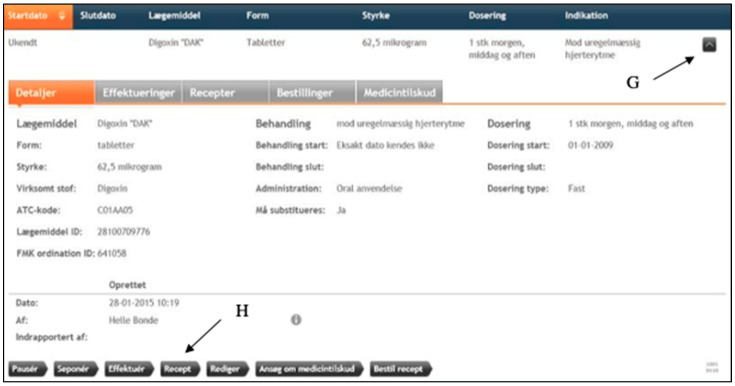
Current prescription page. G Unfold ‘current prescription’ and click on H ‘prescription.

**Figure A4:**
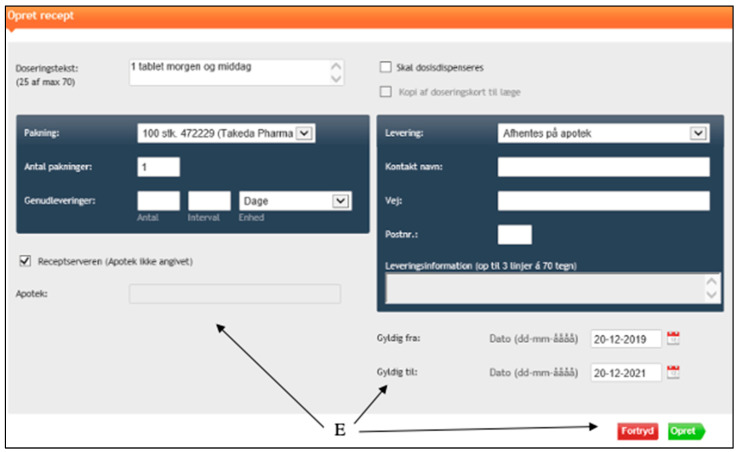
The prescription page where to E ‘create a prescription’ and fill in the relevant medication information.

**Figure A5:**
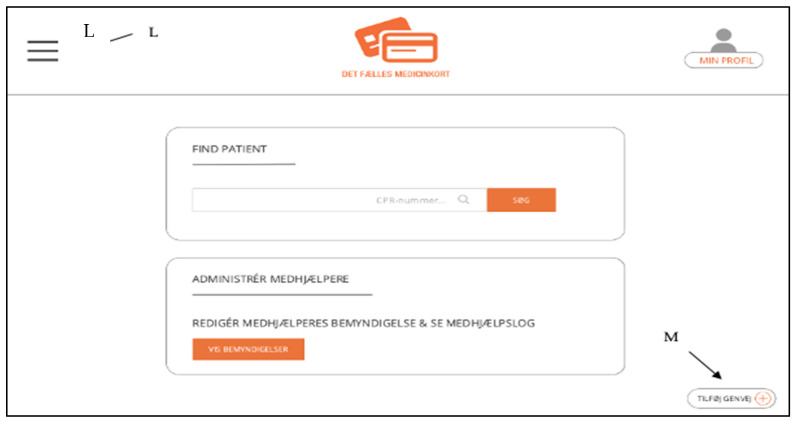
Redesigned frontpage for patient data and entry. L ‘Dropdown navigation menu’ and M ‘Add shortcut’-button M.

**Figure A6:**
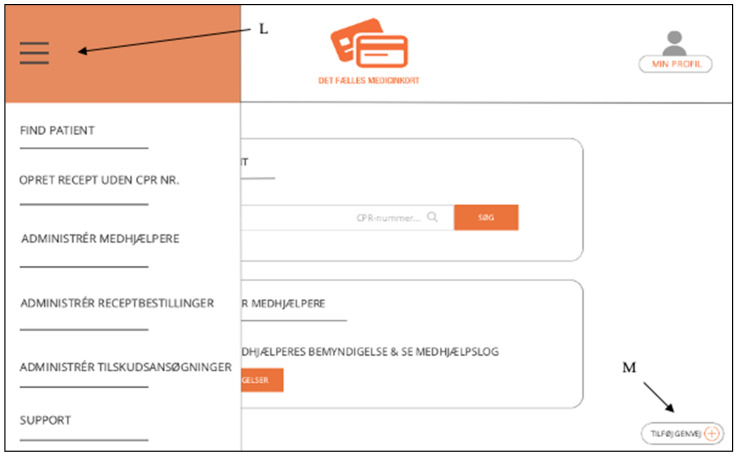
Unfolded L ‘Dropdown navigation menu’ and M ‘Add shortcut’-button M.

**Figure A7:**
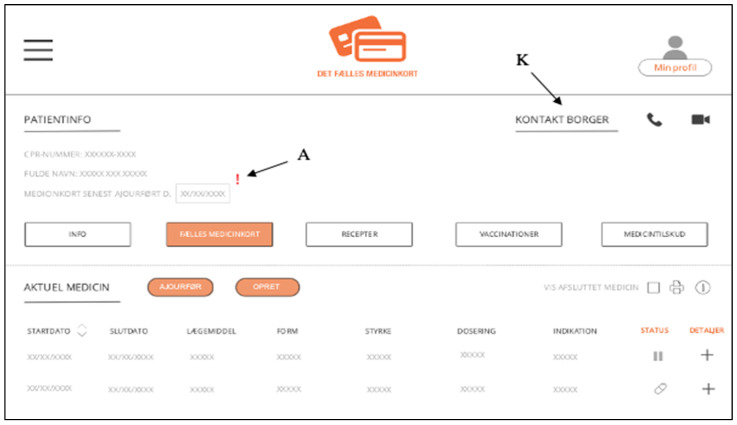
Notification appears A ‘Last updated’-field. K ’Contact citizen/patient’.

**Figure A8:**
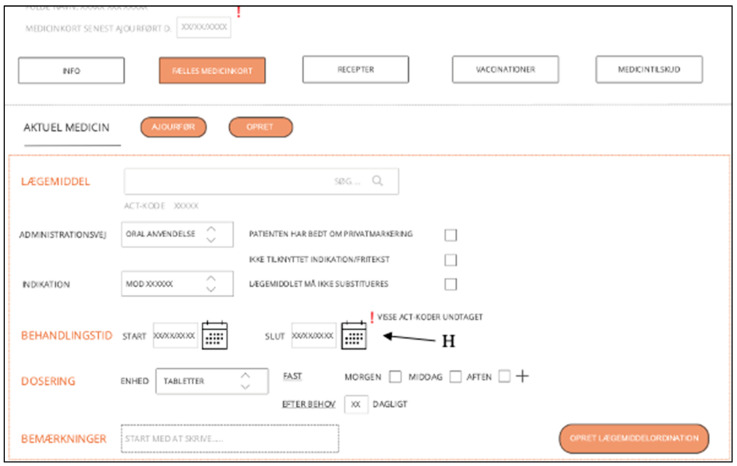
Requires adding end date to the prescription in the H ‘End date’-field.

**Figure A9:**
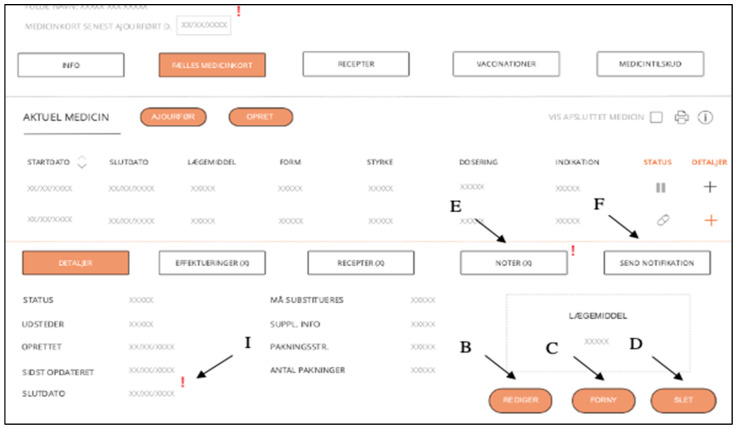
Different prescription possibilities buttons added, B ‘Edit’-button, C ‘Renew’-button, D ‘Delete’-button, and ‘Note’-button E, ´Send notification´-button F and ´Add end-date-button I.

**Figure A10:**
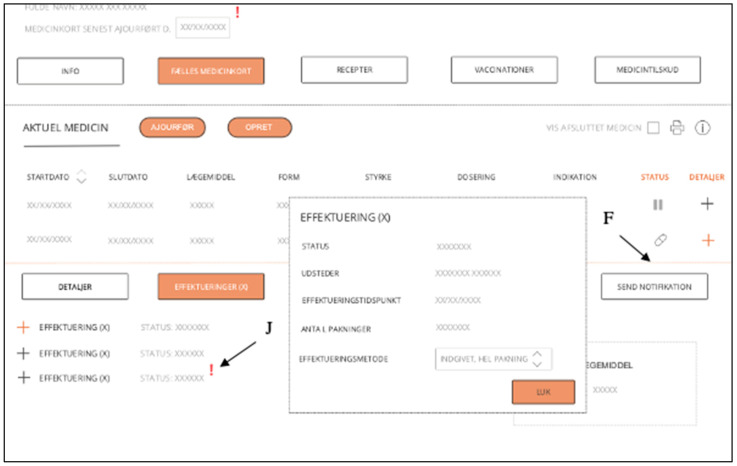
Ensures communication to a general practitioner with F ‘Send notification’-button and J ‘Irregularities in effectuation’-function.

**Figure A11:**
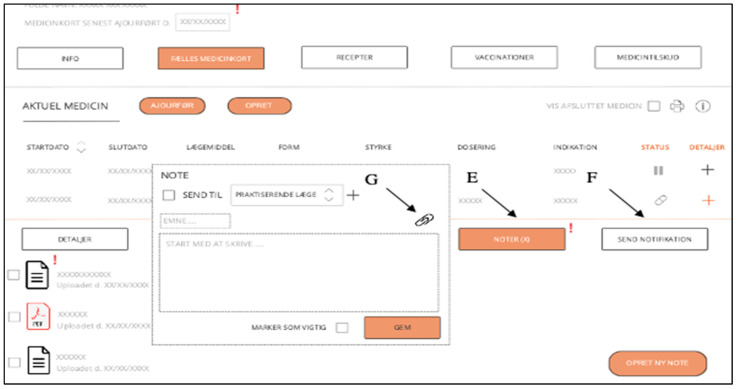
Possibility to add more notes through E ‘Note’-button with attached files through G ‘Attach file/document’-button. If you want to send information to a general practitioner, press F ’send notification’.

**Figure A12:**
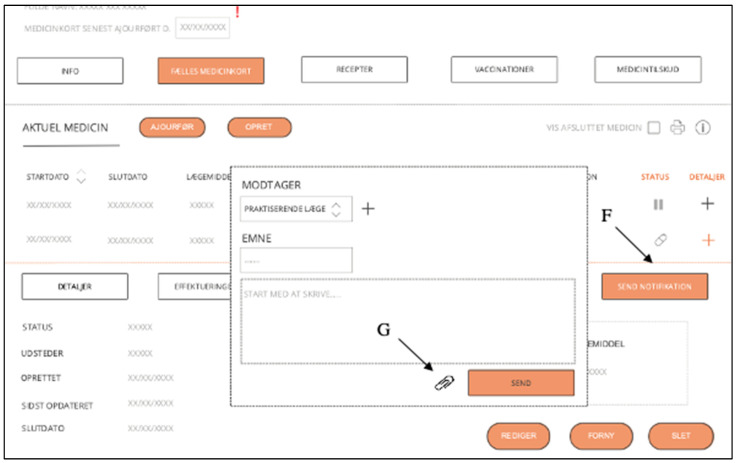
The G ‘Attach file/document’-button’ is also accessible directly through ’send notification’-button F.

## Data Availability

The data presented in this study are not publicly available.

## Appendix B. Identified Codes and Themes

Identified codes through thematic analysis formed the foundation for the primary five themes that later became the foundation for developing the prototype. The interviews were conducted during October 2020.

**Code**	**Count**	**Theme**
“Updating”	19	The SMR does not get updated
“Responsibility”	18	Uncertainties as to who has the primary responsibility of updating the SMR
“The system”	45	The system is too complex, time-consuming, and lacks an intuitive approach
“Familiarity”	25	There is an absence of familiarity with the SMR from the patient’s side, which makes their overview of actual medicine non-efficient
“Involvement”	13	There is a need for involvement by other relevant medical professionals and/or the patients

## Appendix C. Codes and Key Citations from Interviews

Key citation from respondents. The quotes were organized according to codes and selected as they reflect the overall attitude identified during coding from the initial transcripts of the interviews.

**Updating**	**Responsibility**	**The System**	**Familiarity**	**Involvement**
**Nurse 1:** *“I think that the task of reviewing the patient’s medicine lays on the doctor. The doctor should also be the one who removes what needs to be removed.”*	**Pharmacist 1:** *“…The concept of both sectors being able to see each other’s work is a genius thought, but this is dependent on the user—the patient. If the user does not act as intended on the platform, then it is a challenge..”*	**Doctor 3:** *“The thing with SMR is that the concept works well, and it is really handy that it is available on every device. The problem with SMR is the integration with the health platform. This is horrible.”*	**Patient 2:** *“I know SMR because back in the day, I participated in a survey about it and what features it should have; Now I use it, and I think it works pretty well.”*	**Doctor 2:** *“It may be linked to age and tech flair, but I think a lot of patients will benefit from more involvement… I think it could create dialogue and also be easier for doctors to clean up old medicine or update the SMR.”*
**Nurse 2:** *“It is a requirement that the patients get a medical note. We can’t even print this note because the doctors technically have not approved it in SMR. This affects us and makes it difficult for us to do our jobs.”*	***Pharmacy technician 1:*** *“I think that a part of the issue is that the responsibility is complex in some matters. I mean, you need to consider, when a patient gets prescribed medicine, it is usually not just one person, but several medical professions who are a part of this process…”*	**Nurse 1:** *“I think the system works well, and I just think there are too many clicks. There are a lot of working processes if I have to edit a prescription of insulin…”*	**Nurse 3:** *“Not a lot of those who use SMR actually have an overview over their medicine. It is rare that I experience people using it—including the younger patients.”*	***Pharmacy technician 1:*** *“An approach to this could be to make it more patient-oriented. Because this needs to be optimized for them as well, right?... They play a big role in making SMR run smoothly…”*
**Doctor 1:** *“I think it is great that I am able to see an overview of the prescribed medicine and have a dialogue about what works and what don’t.. before, all the paperwork was impossible to structuralize…”*	**Doctor 2:** *“There has been a battle between doctors and hospitals—cause who’s responsibility is it?...”*	**Pharmacist 1:** *“…SMR is so user and culturally dependent… I also talked with doctors who, as an unwritten rule, don’t want to edit on other doctors work.”*	**Doctor 1:** *“It is so different… I have patients who know exactly how much and what they take—and also their household… Others struggle to form an understanding of”*	**Pharmacist 2:** *“Why not make the dialog with the patients more open, to sort out their medicine and also reduce the medicinal waste…”*
